# Do sex differences in CEOAEs and 2D:4D ratios reflect androgen exposure? A study in women with complete androgen insensitivity syndrome

**DOI:** 10.1186/s13293-017-0132-z

**Published:** 2017-04-12

**Authors:** Judy van Hemmen, Peggy T. Cohen-Kettenis, Thomas D. Steensma, Dick J. Veltman, Julie Bakker

**Affiliations:** 1 Netherlands Institute for Neuroscience, Amsterdam, The Netherlands; 2 Department of Medical Psychology, Neuroscience Campus Amsterdam, VU University Medical Center, Amsterdam, The Netherlands; 3 Department of Psychiatry, VU University Medical Center, Amsterdam, The Netherlands; 4 GIGA Neurosciences, University of Liège, Avenue Hippocrate 15, 4000 Liège, Belgium

**Keywords:** Otoacoustic emissions, Digit ratios, 2D:4D, Sex hormones, Androgens, Testosterone, Sex chromosomes, Sexual differentiation, Complete androgen insensitivity syndrome, CAIS

## Abstract

**Background:**

Studies investigating the influence of perinatal hormone exposure on sexually differentiated traits would greatly benefit from biomarkers of these early hormone actions. Click-evoked otoacoustic emissions show sex differences that are thought to reflect differences in early androgen exposure. Women with complete androgen insensitivity syndrome (CAIS), who lack androgen action in the presence of XY-chromosomes, enabled us to study the effect of complete androgen inaction. The main goal was to investigate a possible link between click-evoked otoacoustic emissions and effective androgen exposure and, thus, whether this can be used as a biomarker. In addition, we aimed to replicate the only previous 2nd vs 4th digit-ratio study in women with CAIS, because despite the widely expressed criticisms of the validity of this measure as a biomarker for prenatal androgen exposure, it still is used for this purpose.

**Methods:**

Click-evoked otoacoustic emissions and digit ratios from women with CAIS were compared to those from control men and women.

**Results:**

The typical sex differences in click-evoked otoacoustic emissions and digit ratios were replicated in the control groups. Women with CAIS showed a tendency towards feminine, i.e., larger, click-evoked otoacoustic emission amplitudes in the right ear, and a significant female-typical, i.e., larger, digit ratio in the right hand. Although these results are consistent with androgen-dependent development of male-typical click-evoked otoacoustic emission amplitude and 2nd to 4th digit ratios, the within-group variability of these two measures was not reduced in women with CAIS compared with control women.

**Conclusions:**

In line with previous studies, our findings in CAIS women suggest that additional, non-androgenic, factors mediate male-typical sexual differentiation of digit ratios and click-evoked otoacoustic emissions. Consequently, use of these measures in adults as retrospective markers of early androgen exposure is not recommended.

## Background

Prenatal androgen exposure plays an important role in the development of sex differences in a variety of neural and behavioral characteristics (e.g., reviewed in [1]). To investigate potentially permanent, i.e., organizational, effects of prenatal androgen exposure in humans, a prospective study design including direct measurements of fetal testosterone levels, preferably at several gestational time points, would be ideal. However, this type of study design is expensive, time consuming and, above all, incurs fetal risks and thus is unethical. A desirable alternative would be to use retrospective measures to index prenatal testosterone exposure and action in both sexes.

Otoacoustic emissions (OAEs; e.g., reviewed in [2]) might represent such an indirect marker because sex differences which potentially reflect sex differences in early androgen action have been demonstrated. OAEs are sounds measurable in the external ear canal that originate from the cochlea as a by-product of the cochlear amplification mechanism [3–5]. They can occur spontaneously (spontaneous OAEs; SOAEs) or in response to acoustic stimuli such as clicks (click-evoked OAEs; CEOAEs). Previous studies showed that women had more and stronger SOAEs and larger CEOAE amplitudes than men ([6, 7]; for review see [2]). These sex differences were not only present in adults but also were found in neonates and appeared to remain reasonably constant throughout life [8–12].

A direct investigation of the proposed link between prenatal hormone levels and OAEs later in life is complicated in humans because hormonal manipulations are unethical and the incorporation of direct fetal hormone level measurements in a study design is challenging. To date, OAEs have not been related to fetal testosterone levels across development. Another approach used in examining the validity of OAEs as a marker of early androgen exposure is to study disorders/differences of sex development (DSD) in which the development of chromosomal, gonadal, and/or anatomical sex is atypical [13]. In 46,XX women with congenital adrenal hyperplasia (CAH), a condition characterized by excessive prenatal androgen production, the number of SOAEs was lower (masculinized) compared to control women. However, this was found in the right ear only, and no differences were observed in the CEOAE amplitude of either ear [14].

A DSD of particular interest when studying androgenic effects is complete androgen insensitivity syndrome (CAIS). Women with CAIS have a 46,XY karyotype, but no effective androgen action due to non-functional androgen receptors caused by genetic mutations in the androgen receptor gene. This leads to a female phenotype despite testosterone levels, produced by their abdominal testes, that are within or above the male range [15, 16]. Women with CAIS thus provide a unique opportunity to study whether the reported sex differences in OAEs are likely to originate from differences in androgen action or other factors that might influence sexual differentiation, such as sex chromosome configuration.

To date, the single OAE study that has been performed in women with CAIS found no differences between control subjects and women with CAIS. Both the number of SOAEs and amplitude of CEOAEs were equivalent in CAIS and control women [14]. It should be noted that in this study, the typical sex difference in CEOAE amplitude was not replicated in the control groups and the sample sizes were very small, also for the control groups (7 women with CAIS, 13 control women, 10/11 control men). Also, groups were not matched for age and ethnic background. Therefore, no firm conclusions can be drawn from this study about sex differences in CEOAEs and whether any differences observed can be attributed to differential androgen action. Because strong evidence for a link between early androgen exposure and CEOAEs is still lacking, the primary objective of the present study was to investigate the proposed association between androgens and CEOAE amplitude by comparing women with CAIS to control men and women.

The 2nd digit to 4th digit (2D:4D) ratio, which is the length of the 2nd digit (index finger) relative to that of the 4th digit (ring finger), is another measure that has been proposed to reflect sex differences in prenatal androgen exposure. The 2D:4D ratio is generally larger in women than in men ([17]; for meta-analysis, see [18]). However, the validity of this measure as a marker of prenatal androgen exposure has been criticized. Two digit-ratio studies [19, 20] attempted to measure fetal androgen levels through amniocentesis, and their results were inconclusive. The 2D:4D ratio showed a negative correlation with testosterone content measured in amniotic fluid in newborn girls, but not in boys [19]. Also, at age 2 years, a negative correlation was observed between 2D:4D ratio and the ratio of testosterone to estradiol (T:E) measured in amniotic fluid [20]. However, it should be noted that the T:E ratio in the latter study [20] was based on a sample of 29 children, boys and girls combined, and given the small sample and sex differences in all three variables measured (testosterone, estradiol, 2D:4D) likely was spurious. In addition, a recent meta-analysis found no associations between functional androgen receptor (AR) gene variants (CAG and GGC repeat-length polymorphisms) and 2D:4D ratio [21], providing evidence against major AR gene-related effects (for a discussion of additional evidence and arguments against AR gene-related effects on 2D:4D, see [21]).

Digit-ratio studies in DSDs suggested a lower, i.e., more “masculine,” digit ratio in women with CAH than in control women for either the right hand [22, 23] or for both hands [24, 25], although a “feminine” digit ratio in CAH was reported in yet another study [26]. Individuals with Klinefelter syndrome, who have 1 Y- and 2 or more X-chromosomes and might have reduced fetal androgen exposure, showed a higher, i.e., “feminized”, digit ratio than control men [27]. A study in 16 women with CAIS showed that their digit ratios were feminized, which, along with the majority of findings in DSDs, points to a link between fetal androgen exposure and the 2D:4D ratio [28]. However, some important caveats have been discussed by the authors of this study [28] and in a commentary on this paper [29]. First, the effect size of the difference between women with CAIS and control men was only moderate, and second, the variability in digit ratios was not smaller in women with CAIS than in either the male or female control subjects. If fetal androgen exposure alone determines 2D:4D ratios, then, one would expect less variability in women with CAIS because they are completely insensitive to any androgen actions. This has led to the conclusion that 2D:4D digit ratio is partially determined by fetal androgen exposure but that the relationship is too small to use digit ratio as a reliable marker for fetal androgen exposure in individual subjects (as cited in [28]). Regardless of this conclusion and other important critical arguments against AR-gene-related effects (summarized by [21]), numerous investigators continue to use the digit ratio as an index of fetal androgen action. The study by Berenbaum et al. [28] included a sample of only 16 women with CAIS, which is a reasonable sample considering the rareness of the syndrome. However, the statistical power of the comparisons made with control subjects was quite low. For this reason, we sought to replicate this study using a larger sample of CAIS women as well as control men and women. Thus, this is the first study analyzing both CEOAE and 2D:4D in women with CAIS.

## Methods

### Participants

Subjects whose data were used in the present study were Caucasian, did not report having a male twin, and had a heterosexual orientation. A heterosexual orientation was defined as an androphilic orientation, i.e., attraction to men, in control women and women with CAIS, and a gynephilic orientation, i.e., attraction to women, in control men (sexual orientation score >5 on the Dutch version of the Klein Sexual Orientation Grid ([30]; adapted from [31])). Note that we used Caucasian as selection criterion, because all women with CAIS were Caucasian, and ethnicity affects CEOAEs and 2D:4D digit ratios. Not all subjects met all our additional inclusion criteria for both the CEOAE and digit ratio analyses. Therefore, further details of the participant groups will be described for both analyses separately.

For the CEOAE analyses, subjects were divided into four groups: women with CAIS, control men, and, because hormonal contraceptives might influence the CEOAE amplitude [32, 33], two groups of control women, those using oral contraceptives (OC-women) and normally cycling (NC-)women not using any hormonal contraceptives. To minimize effects related to handedness [7], all subjects had a right-hand preference for writing and handedness scores of ≥+7 on the Dutch Handedness Inventory ([34]; range −10 = extreme left-handed, +10 = extreme right-handed). All control women were pre-menopausal and not pregnant. NC-women were not tested during the first days of the follicular phase (menses), when sex hormone levels are low, but otherwise could be tested any time during the remainder of their cycle. OC-women also were tested only once and only on a day when they were taking doses of OC, i.e., not during the first week of the cycle when OC is not taken. All OC-women used a monophasic OC, containing ethinylestradiol and levonorgestrel (20/100 μg (*N* = 2), 30/150 μg (*N* left ear = 27, *N* right ear = 30) or 50/125 μg (*N* = 2)). The data for both ears were excluded for all subjects exposed to high levels of noise in the 24 h preceding the measurement and from subjects who reported having a hormonal disorder or taking hormonal medication that might influence sex hormone levels (with the exception of oral contraceptives for OC-women and hormone replacement therapy in women with CAIS). When severe ear problems were reported or measurements were invalid (see “CEOAE” in methods section), the data from that particular ear were excluded from the analyses. These criteria resulted in a total of 125 usable measurements of the left and 132 usable measurements of the right ear in all groups combined, see Table 1 for *N*s and age per group.

**Table 1:** Sample characteristics, CEOAE amplitude, and 2D:4D ratio per group

	CAIS	Control women	Control Men	*F*/*χ* ^2^	*p*	*η* ^2^
	M/Mdn (SD/IQR)	M/Mdn (SD/IQR)	M/Mdn (SD/IQR)			
CEOAE left						
*N*	18	74	33			
Age^a^	27.80 (18.11)	27.29 (13.19)	28.30 (19.11)	0.937	0.626	
Amplitude (dB SPL)	10.96 (2.72)	12.85 (4.14)	9.42 (4.19)	8.789	**<0.001**	0.126
CEOAE right						
*N*	18	77	37			
Age^a^	27.80 (18.11)	27.66 (17.76)	28.30 (19.60)	0.341	0.843	
Amplitude (dB SPL)	12.48 (2.94)	12.85(4.36)	10.23 (3.77)	5.357	**0.006**	0.077
2D:4D ratio left						
*N*	21	156	129			
Age^a^	27.65 (23.9)	27.00 (14.0)	26.82 (14.8)	0.655	0.721	
Ratio^a^	0.965 (0.037)	0.980 (0.037)	0.963 (0.045)	13.565	**0.001**	0.044
2D:4D ratio right						
*N*	20	154	126			
Age^a^	27.80 (23.0)	27.00 (12.8)	26.91 (15.0)	1.720	0.423	
Ratio^a^	0.978 (0.038)	0.976 (0.046)	0.956 (0.043)	22.819	**0.000**	0.076

Note: Statistics from parametric tests are expressed in M = mean, SD = standard deviation, *F*. Statistics from non-parametric tests are expressed in Mdn = median, IQR = interquartile range (these are more appropriate statistics for non-parametric data), and *χ* ^2^. Bold *p* values represent a significant (*p* < 0.05) main effect of group. *η* ^2^ = partial eta squared values for the three group comparisons.

^a^Non-parametric tests were used for statistical analysis of this variable

For the digit-ratio analyses, the use of OCs was not a factor of interest; therefore, the data of only three groups were used for this part of the study, women with CAIS, control men, and NC-women. The latter group will be referred to as “control women.” It is important to mention that controls were completely unrelated to the women with CAIS. The 2D:4D ratios of 7 left and 13 right hands were excluded from the analyses because of reported digit or hand trauma with a possible influence on digit length, or due to technical problems with the scan of the hand. To increase the size of the control groups, data from 88 control men and 109 control women from a previous digit ratio study, all heterosexual and 18 years and older, were added to the data acquired for the current study (see [35] for further details). This resulted in a total of 306 usable left and 300 usable right-hand 2D:4D ratios for all groups combined (see Table 1 for *N*s and age per group and per hand).

All women with CAIS had gender identity scores in ranges comparable to reference groups of healthy women, which was assessed with a Gender Questionnaire (Callens et al. submitted; adapted from [36]) with six questions added to the original questionnaire to cover the criteria for a gender identity disorder based on the Diagnostic and Statistical Manual of Mental Disorders [37]. The diagnosis of CAIS was based on clinical characteristics in all 22 women with CAIS included in the analyses. Furthermore, in contrast to the Berenbaum et al. study ([28], a mutation analysis of the androgen receptor (AR) gene using genomic DNA was performed. In 14 participants, the clinical diagnosis was confirmed with a mutation in the AR-gene, in 7 participants, an unclassified variant of the AR-gene mutation was found, and in 1 participant, the result of the analysis was inconclusive. All women with CAIS were gonadectomized and, with one exception, took hormone replacement therapy (HRT) to compensate for the lack of gonadal sex hormone production. HRT consisted of orally, transdermally, or subcutaneously administered estradiol (*N* = 15), a combination of estradiol and dydrogesteron (*N* = 3), conjugated estrogens (*N* = 2), or a monophasic oral contraceptive containing ethinylestradiol and levonorgestrel (*N* = 1).

Women with CAIS were recruited from the databases of the VU University Medical Center Amsterdam and the Erasmus University Medical Center-Sophia Children’s Hospital Rotterdam, as well as from the support group DSDNederland. The majority of these women with CAIS were recruited for a larger neuroimaging study (e.g., described in [38]). Control subjects were recruited from employees of the VU University Medical Center and by using flyers and advertisements in a local newspaper.

### CEOAEs

CEOAEs were recorded using the ILO288 Echoport (Otodynamics Ltd., UK) connected to a laptop with EZ-screen software. Prior to a testing session, the probe was calibrated using a calibration cavity. For each participant, a single-use probe tip was selected with an appropriate size to best fit and seal the external ear canal. Participants were tested in a quiet room while seated and were instructed not to move and particularly to avoid jaw action, swallowing, and vocalizations to prevent cable-rub and ear canal noise during CEOAE recording. CEOAEs always were measured first for the right ear. The QuickScreen (non-linear) mode was used with a 2.5 to 12.5-ms post-click response window. The click stimulus level was set at 80 dB sound-pressure level (SPL) (*M* = 80.23, SD = 0.80), and 250 responses with a noise level below 6 mPa were recorded per ear. CEOAEs were recorded in five frequency bands (1000, 1414, 2000, 2828, and 4000 Hz), but only the absolute CEOAE response, which relates to the total OAE energy recorded across the frequency range, was used for the analyses. A recording was considered valid when showing a minimum amplitude of 1 dB SPL and a whole band reproducibility of at least 70% based on two independently recorded waveforms.

### Digit ratios

For the present study, high-resolution (600 × 600 dpi) digital scans of the hands were made using a Canon imageRUNNER ADVANCE. The left and right hand were scanned separately. Participants were asked to remove rings and gently press their hand on the glass of the scanning device while straightening and slightly spreading their fingers. Prior to scanning, a photocopy of the hand was made to ensure correct positioning of the hand and quality of the photocopy. Two independent raters, unaware of the participant’s group membership, measured the digits using AutoMetric (version 2.2; deBruine, 2006), a program designed to measure hand properties from digital images. The raters manually marked the fingertip and the middle of the basal crease on the ventral surface of the 2nd and 4th digit. The software used these landmarks to automatically calculate the length of the 2nd and 4th digit in pixels and the 2D:4D ratio for each hand. The intra-class correlation coefficient (two-way mixed effects model, type absolute agreement) was >0.997 for all four (left and right 2nd and 4th) digits vs 0.916 and 0.775 for the calculated digit ratios of the left and right hand, respectively, indicating a high interrater reliability. Therefore, the average of each digit ratio from the two raters was calculated and used for the analyses. See [35] for the methods used for the additional control subjects.

### Data analysis

Statistical analyses were performed with IBM SPSS Statistics for Windows, Version 22 (IBM Corp., Armonk, NY, USA). First, we used a *t* test to assess whether OC- and NC-women showed differences in CEOAE. Because no differences were found (see “Results” section), OC- and NC-women were grouped for subsequent analyses in order to have a larger sample size for the women control group. We used one-way analyses of variance (ANOVA), followed by Bonferroni post hoc pairwise comparisons, to analyze differences in left and right CEOAE amplitudes between control men, control women, and women with CAIS. Effect sizes for pairwise comparisons of the CEOAE data are expressed in Cohen’s *d*, with effect sizes of 0.2, 0.5, and 0.8 referring to small, medium, and large differences, respectively [39]. Cohen’s *d* was calculated as the mean difference between the two groups divided by the pooled standard deviation which is the square root of the weighted sum of the individual variances for the two groups. Differences in variance between women with CAIS and the control groups were assessed with pairwise Levene’s tests.

Kruskal-Wallis and post hoc Mann-Whitney *U* tests were used for the between-group analyses of the left and right 2D:4D ratio, because the right-hand data of women with CAIS and control men were not normally distributed. Effect sizes for pairwise comparisons of the 2D:4D ratio data are expressed in *d*, as calculated from *r* (*Z* statistic divided by the square root of the combined sample size of the two groups). Differences in variance between women with CAIS and the control groups were assessed with pairwise Levene’s tests.

For all analyses, results were considered significant at a *p* < 0.05 threshold. Post hoc pairwise test statistics were corrected for multiple comparisons using a Bonferroni correction.

## Results

### CEOAEs

**Fig. 1:**
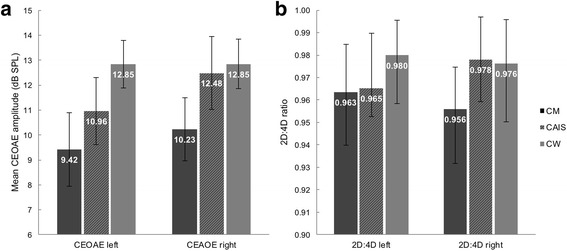
**a** Mean (+95% confidence interval) CEOAE amplitude in the left and right ear per group. **b** Median (+IQR) left and right-hand 2D:4D ratio per group. *CM* control men, *CW* control women

The CEOAE amplitudes of NC-women (left ear *M* = 12.68, SD = 4.08; right ear *M* = 12.93, SD = 3.67) and OC-women (left ear *M* = 13.07, SD = 4.26; right ear *M* = 12.74, SD = 5.16) showed no significant differences (*p* = 0.690 and 0.859 for left and right ear, respectively). Data from both OC- and NC-women were grouped together to have one large group of control women in the ANOVA to assess between-group differences. This ANOVA showed a significant effect of group on CEOAE amplitude in both ears (Table 1). Figure 1a presents the mean left and right-ear CEOAE amplitude for each group with error bars representing the 95% confidence interval. Post hoc pairwise comparisons (Table 2) revealed the expected sex differences between control men and control women [2] in both ears, with greater mean CEOAEs in control women and medium-to-large effect sizes. The greater mean right-ear amplitude in women with CAIS than in control men was however not significant (*p* = 0.163). The other post hoc comparisons also did not reveal significant between-group differences. Variances for both the left and right-ear CEOAE amplitudes did not differ between women with CAIS and the control groups (all *p*s > 0.05 even before applying a Bonferroni correction for multiple comparisons).

**Table 2:** Post hoc tests, effect sizes, and differences in variance for CEOAE amplitude and 2D:4D ratios

	Left			Right		
	*p*	Cohen’s *d*	Levene’s *p*	*p*	Cohen’s *d*	Levene’s *p*
CEOAE amplitude (dB SPL)						
Control men vs control women	**<0.001**	0.824	1.000	**0.005**	0.642	0.399
Control men vs CAIS	0.571	0.436	0.285	0.163	0.666	1.000
Control women vs CAIS	0.220	0.540	0.273	1.000	0.097	0.174
2D:4D ratio^a^						
Control men vs control women	**0.001**	0.438	0.058	**0.000**	0.574	0.469
Control men vs CAIS	1.000	0.087	0.152	**0.043**	0.414	0.440
Control women vs CAIS	0.411	0.225	0.513	1.000	0.002	0.606

Note: Bold *p* values represent a significant (*p* < 0.05) between-group difference after applying a Bonferroni correction for multiple comparisons.

^a^Non-parametric tests were used for statistical analysis of this variable

### Digit ratios

For both the left and right-hand 2D:4D ratios, a main effect of group was found (Table 1). Median digit ratios and the interquartile range (IQR) are displayed in Fig. 1b. Post hoc comparisons showed the typical significant sex difference in both hands [18], with higher 2D:4D ratios in control women than men and medium effect sizes. In the right hand, women with CAIS also showed a higher digit ratio than control men, with a small-to-medium effect size. No other comparisons reached significance. Levene’s tests showed no differences in variance between women with CAIS and control groups in digit ratios of either hand (all *p*s > 0.05 even before applying a Bonferroni correction for multiple comparisons).

## Discussion

The main objective of the present study was to replicate the previous CEOAE study [14] and the previous digit-ratio study [28] in women with CAIS in order to further assess the use of each of these variables as indirect indicators of fetal testosterone action. The results from each measure will be discussed separately.

### CEOAEs

In line with previous studies, (e.g., reviewed in [2]), sex differences with medium-to-large effect sizes were found in both ears, with higher amplitudes in control women than in men. Although none of the differences between women with CAIS and the control groups reached statistical significance, there was a trend towards a female-typical CEOAE amplitude in the right ear of women with CAIS. A similar tendency for female-typical CEOAEs in women with CAIS was, however, not found in the left ear.

Although conclusions based on the right-ear tendency towards female-typical CEOAEs in women with CAIS should be drawn with great caution, the lack of a significant difference between women with CAIS and control men is likely the result of low statistical power caused by the small sample of women with CAIS, along with the strict statistical threshold that was applied (i.e., two-tailed tests and corrections for multiple comparisons). Taking this into consideration, the direction of the effect is in line with the hypothesis that low prenatal androgen action is associated with stronger, female-typical, CEOAEs.

Because the only previous OAE study in DSDs failed to replicate the typical sex difference in CEOAEs [14], an interpretation of those results and comparison with the results from the present study is difficult. However, a sex difference, with medium-to-large effect sizes*,* was replicated in SOAEs, which in general show a large correlation with CEOAEs [40], suggesting an overlap in mechanisms responsible for the production of these auditory characteristics. Accordingly, females with CAH showed a masculinized number of SOAEs in the right ear [2]. Although the number of SOAEs in women with CAIS was not significantly different from control men or women, the effect sizes revealed a deviation from control men (*d* = 0.56) in the direction of control women in the left ear. This is in the same direction, although with a slightly smaller effect size, as the female-typical tendency found in right-ear CEOAEs in the present study.

The authors of the previous OAE study in DSDs [14] suggested that OAEs “may prove useful as bioassays for assessing early brain exposure to androgens” (as cited in [14]), although those authors did emphasize the need for replication. This statement is only statistically supported by the right-ear SOAE results of the CAH group in their study, and it should be noted that a chance finding cannot be ruled out when taking into account the small sample sizes for DSD and control groups in the study by Wisniewski et al. [14]. In the present study, we have further assessed this potential association between CEOAEs and fetal androgen exposure by examining the variability of this measure in women with CAIS. If effective androgen action, prenatally and/or later in life, were a main determinant of observed sex differences in CEOAE amplitude, then women with CAIS would show a much smaller variability in CEOAE amplitudes due to their complete androgen inaction as opposed to the more variable androgen exposure to be expected in control men as well as women. However, in the current study, the variability of CEOAE amplitude for both ears was not reduced in women with CAIS. Therefore, although the right-ear results point to a role for effective androgen exposure in CEOAE amplitude and the left-ear results are inconclusive, the finding that CEOAEs in women with CAIS were as variable as in both control groups suggests that factors in addition to fetal androgen exposure influence the CEOAE amplitude measured in adulthood.

Previous studies have addressed the potential influence of circulating hormone levels in adulthood on OAEs. CEOAE amplitudes in control men correlated with seasonal variation in testosterone levels [41]. However, these effects cannot explain the variability of CEOAE amplitudes in women with CAIS, as they are insensitive to androgens throughout life. In addition, small changes related to the menstrual cycle have been reported for SOAEs [42–45], with some of these studies suggesting a positive association with high estrogen phases of the menstrual cycle. Estrogen receptors have been found in the adult human cochlea [46], and thus, estrogens could affect cochlear function. With one exception, all women with CAIS included in our CEOAE analyses used HRT containing estradiol. Therefore, the tendency towards female-typical CEOAE amplitude in the right ear might result from high estradiol levels in combination with the absence of androgen effects. Estrogen actions also could explain the variability in CEOAE amplitude in women with CAIS, because they have normally functioning estradiol receptors and variable estradiol levels from HRT.

Previous studies also have suggested that oral contraceptives might have a defeminizing or masculinizing influence on CEOAEs [32, 33], although only the study by Snihur and Hampson reported significant effects in support of this hypothesis. Because of this proposed influence, both OC- and NC-women were included in the present study. However, because no CEOAE amplitude differences were found between these groups, we grouped both samples in one large female control group. Although an investigation of OC-related effects in control women was not the primary aim of the present study, we will briefly discuss this issue because our results differ from the study by Snihur and Hampson [32]. In the present study, the sample sizes were larger and the OC formulation more homogeneous, with all women using a monophasic OC, whereas in the study by Snihur and Hampson only 40% used a monophasic OC and 60% used a triphasic OC with varying progesterone dosages over the cycle. In addition, in the present study, OC-women were tested at a day of OC use, whereas Snihur and Hampson did not take this factor into account. Based on the typical 4-week OC cycle, in which during 1 week no OC is used, in theory, a quarter of the OC using women in the Snihur and Hampson study could have been tested on a day without OC use, and thus could have had much lower estrogen and progesterone levels. Although purely speculative, such a bias could explain the lower CEOAE amplitudes in OC-women in their study. The finding that CEOAEs did not differ between OC- and NC-women in the present study, therefore, argues against the hypothesis proposed by Snihur and Hampson [32], that ovarian-derived estrogen, and not the synthetic ethinylestradiol used in OC formulations, acts to facilitate the cochlear amplifiers. Instead, it is at least possible that the differences found in the previous study were the result of differences in estrogen levels, regardless of their origin.

A limitation of the present study is that potential menstrual cycle-related effects were not controlled for in NC-women. Although in a subgroup of NC-women, who were also included in a larger magnetic resonance imaging study, attempts have been made to perform testing only during the high estrogen phase of the menstrual cycle (around ovulation), serum hormone levels that were obtained in this subgroup revealed that these women were tested throughout different phases of their cycle, although not during menses. In the additional subjects that were tested to increase the sample size for the current study, no blood samples were obtained. Therefore, we tested this new group throughout the menstrual cycle, with the exception of the menstrual phase, to match the initial group of NC-women. In addition, testing NC-women throughout their menstrual cycle (except for the menstrual phase) reduced a potential cycle-related bias, and this group would serve as a more typical control group for women with CAIS, because the latter group used hormone replacement in different dosages. Furthermore, exclusion of the menstrual phase would be a better match to women with CAIS, who continuously use their HRT, and OC-women, who were not tested during their week without OC intake.

Although in the present study the sample size of women with CAIS is twice as large as in the study by Wisniewski et al. [14], thereby increasing statistical power, the sample is still relatively small. It is challenging to acquire a sufficient amount of data from women with CAIS given the low incidence rate of the syndrome, estimated to be between 1:40,800 and 1:99,000 for all subtypes (mild, partial, and complete) of AIS combined [47]. Therefore, this methodological limitation, which also applies to the digit ratio data, is difficult to overcome.

### Digit ratios

The previously reported sex difference in the 2D:4D ratio, with larger digit ratios in women than men [18], was replicated in the control groups, with moderate effect sizes, in line with previous studies [48]. In addition, our results in women with CAIS relative to the control groups were similar to the findings presented by Berenbaum et al. in 2009 [28]. The female-typical digit ratios in women with CAIS, which are in line with androgen-related effects on digit ratios, were replicated in the right hand. In the present study, this result reached significance while applying a more stringent statistical threshold (two-tailed tests and correction for multiple comparisons) than what was used in the previous study, thereby reducing the risk of a type I error. The small-to-medium effect size for the difference between control men and women with CAIS was only slightly smaller than in the study by Berenbaum et al., which was 0.61 for the right hand. In the present study, this female-typical effect was not observed for the left hand, which seems to be explained by a lower left-hand 2D:4D ratio in women with CAIS than in the study by Berenbaum et al. However, after careful inspection of the individual digit ratios from this previous study (Fig. 1, [28]), there was one woman with CAIS who had a rather extreme high left-hand digit ratio, which can explain the higher mean left-hand digit ratio of women with CAIS in that study. It is likely that a median for that same data set, which is insensitive to extreme values, would be lower and more similar to the median in our study.

It has been suggested that the sex difference in digit ratio is generally larger in the right hand [18]. The current results suggest a slightly larger effect size for the sex difference in the right relative to the left hand. It has been proposed that this could be the result of a greater sensitivity of the right-hand digit ratio to prenatal testosterone [17], which might explain why women with CAIS showed no significant deviations from either of the control groups in the left hand. However, this lack of significant differences prevents us from drawing firm conclusions regarding the left-hand digit ratios in women with CAIS.

The analysis of the variability in digit ratios provides additional information regarding the magnitude of the potential influence of fetal androgen action on digit ratios from both hands. Women with CAIS did not show a reduced variability in digit ratios, which would be expected if androgen action alone determined digit dimensions. This is also a replication of the findings from the previous digit ratio study in women with CAIS [28], and again indicates that variations in digit ratios reflect not only variations in prenatal androgen action but also are under the influence of additional factors. In line with these findings, a meta-analysis of digit ratio studies showed that the magnitude of the digit ratio sex difference is smaller than the magnitude of the sex difference in amniotic testosterone levels [18]. Furthermore, classification of individual men and women according to their digit ratios was not very reliable [28]. Finally, it should be noted that amniotic testosterone levels might not tell us much about localized androgen levels in the fetus (i.e., in digits vs brain) at specific time points in development.

## Conclusions

The present observation that mean right ear CEOAE amplitudes as well as mean right hand 2D:4D digit ratios in CAIS women resembled values obtained in control women as opposed to control men point to a possible contribution of fetal androgen action to male-typical development of these two variables. Even so, our finding that the variability in both measures was similar across all three groups argues against the notion that differences in CEOAE amplitudes and digit ratios solely reflect differences in fetal androgen exposure, or in androgen exposure later in life. It might not be surprising that two previous studies measuring both digit ratios and CEOAEs or SOAEs in the same subjects did not find an association between these two biomarkers [48, 49]. Although both authors have provided several possible explanations for their finding, such as different developmental time windows for digit vs cochlear development, the explanation stating that “neither measure exhibits a strong relationship with the underlying prenatal processes” (as cited in [49]) is most in line with current knowledge from both the present and previous studies.

Taken together, the present results, along with results from previous studies, suggest that fetal androgen exposure is one factor contributing to the development of male-typical digit ratios and CEOAE amplitudes. Our present results together with those of Berenbaum et al. [28] strongly suggest that additional non-androgenic factors are also needed to establish the male-typical phenotypes for these variables. Consequently, neither marker can be presumed to provide a reliable indication of prenatal androgen action and using these measures for this purpose is not recommended. Further research is needed to clarify what other factors in addition to fetal androgen action determine male-typical digit ratios and CEOAEs and what their exact timing and mechanisms of action might be.

## Abbreviations

2D:4D: 2nd digit to 4th digit
AR: Androgen receptor
CAH: Congenital adrenal hyperplasia
CAIS: Complete androgen insensitivity syndrome
CEOAE: Click-evoked otoacoustic emission
DSD: Disorder/Difference of sex development
HRT: Hormone replacement therapy
IQR: Interquartile range
NC: Normal cycling
OAE: Otoacoustic emission
OC: Oral contraceptives
SOAE: Spontaneous otoacoustic emission
T:E: Testosterone to estradiol
